# Rituximab Concentration Varies in Patients With Different Lymphoma Subtypes and Correlates With Clinical Outcome

**DOI:** 10.3389/fphar.2022.788824

**Published:** 2022-01-26

**Authors:** Shu Liu, Zhao Wang, Rongxin Chen, Xueding Wang, Xiaojie Fang, Zhuojia Chen, Shaoxing Guan, Tao Liu, Tongyu Lin, Min Huang, He Huang

**Affiliations:** ^1^ Institute of Clinical Pharmacology, School of Pharmaceutical Sciences, Sun Yat-sen University, Guangzhou, China; ^2^ State Key Laboratory of Oncology in South China, Collaborative Innovation Center for Cancer Medicine, Sun Yat-sen University Cancer Center, Guangzhou, China; ^3^ State Key Laboratory of Ophthalmology, Zhongshan Ophthalmic Center, Sun Yat-sen University, Guangdong Provincial Key Laboratory of Ophthalmology and Visual Science, Guangzhou, China; ^4^ Sichuan Cancer Hospital and Institute, Sichuan Cancer Center, School of Medicine, University of Electronic Science and Technology of China, Chengdu, China

**Keywords:** rituximab, pharmacokinetic, outcomes, “double-hit” lymphoma, mantle cell lymphoma

## Abstract

Individual variations in concentrations of rituximab in different B cell non-Hodgkin’s lymphoma subtypes and their relevance to efficacy were still unclear. From 2016 to 2021, a prospective clinical trial was conducted, and 510 samples with 6 uncommon subtypes of B-cell lymphoma were enrolled to examine the pharmacokinetic behaviour of rituximab and its impact on clinical outcomes, including complete response (CR), progression-free survival (PFS) and overall survival (OS). Considerable variability was observed in the rituximab trough concentration in the first cycle (C_1-trough_, 1.16–55.52 μg/ml) in patients with different lymphoma subtypes. Patients with “double-hit” lymphoma (4.01 ± 0.77 μg/ml) or mantle cell lymphoma (MCL; 15.65 ± 16.45 μg/ml) had much lower C_1-trough_ and worse outcomes. Great individual variation in the C_1-trough_ existed among patients with mucosa-associated lymphoma (MALT), and the high C_1-trough_ observed in patients treated with the RB regimen was associated with a better response than was obtained with R-CHOP (38.41 ± 14.13 μg/ml vs 15.49 ± 8.80 μg/ml, *p* = 0.0029). Despite the high aggressiveness of the cancer, Burkitt lymphoma patients receiving intensive chemotherapy had the highest C_1-trough_ (28.85 ± 9.35 μg/ml) and maintained long-term PFS. The C_1-trough_ in patients with mixed, unclassifiable B-cell lymphoma was close to 20 μg/ml, and these patients had acceptable outcomes. Overall, a low rituximab C_1-trough_ was associated with adverse consequences, including persistent progression, early recurrence and a short OS, however, some high-risk factors appeared to be balanced by the presence of a high C_1-trough_. Basal levels of circulating CD19^+^ lymphocytes differed between and within patients with diverse lymphoma subtypes and were negatively correlated with C_1-trough_. Therefore, the traditional doses of rituximab are inadequate for patients with “double-hit” lymphoma and MCL. Increasing the initial rituximab dose according to the disease, high-risk factors and even the baseline CD19^+^ lymphocyte count will be new methods to optimize therapeutic regimens for patients with different lymphoma subtypes.

**Clinical Trial Registration**: [http://www.chictr.org.cn/index.aspx], identifier [ChiCTR1800017001].

## Introduction

Since its initial approval in 1997, rituximab has improved the prognosis of all B-cell derived lymphoproliferative diseases, especially B-cell malignancies (Pierpont et al., 2018), including diffuse large B-cell lymphoma (DLBCL), follicular lymphoma (FL), Burkitt lymphoma, high-grade B-cell lymphoma, mantle cell lymphoma (MCL), mucosa-associated lymphoma (MALT), and chronic lymphocytic leukaemia (CLL), etc (Salles et al., 2017). Treatment with rituximab at a dose of 375 mg/m^2^ is currently recommended for nearly all forms of B-cell malignancies. However, there is no definitive consensus on the optimal dosage or the desired drug concentration for patients with different lymphoma subtypes because the original dose was derived from small phase I/II (Maloney et al., 1994; Maloney et al., 1997) studies that excluded these specific subtypes of patients.

A few pilot studies revealed that the pharmacokinetics data for patients with MCL may different from patients with DLBCL and FL. In Mangel’s study (Mangel et al., 2003), rituximab levels were measured in 20 patients with FL and 6 patients with MCL in an autologous stem cell transplant (ASCT) setting and a trend towards lower rituximab levels in patients with MCL was found. Tran et al. (2010) reported 1 MCL patient had the lowest rituximab concentration compared with patients with DLBCL, FL and CLL. One MCL patient also showed approximately 4-fold higher clearance than patients with DLBCL and FL in Yonezawa’s study (Yonezawa et al., 2019). All these studies are invaluable and inspired us to focus on the variations in pharmacokinetics of rituximab in different B cell lymphoma histological type.

The optimal use of rituximab should be based on biological rationale and on a “concentration-response” relationship. For individuals with chronic lymphocytic leukaemia, the recommended rituximab dose is 500 mg/m^2^, because in that disorder, cells express lower levels of CD20, and the disease is associated with higher levels of free circulating CD20 antigen than other B-cell non-Hodgkin’s lymphoma (O'Brien et al., 2001). Moreover, high variability in patient outcomes after rituximab-based treatment could be partially explained by the individual variation in rituximab concentration that was observed in individuals with FL and DLBCL (Berinstein et al., 1998; Golay et al., 2013; Tout et al., 2017); higher rituximab concentrations were associated with better clinical response, and some patients did not achieve an effective concentration at a dosage of 375 mg/m^2^ (Liu et al., 2020). Furthermore, in Pfreundschuh’s study (Pfreundschuh et al., 2017), elderly males who received an initial dosage of 500 mg/m^2^ rituximab displayed improved progression-free survival (PFS) and overall survival (OS), and other trials (Murawski et al., 2014; Strussmann et al., 2017) in DLBCL confirmed that initial dense-dose rituximab provided a promising response for patients with a poor prognosis. Currently, novel strategies are urgently needed to improve outcomes in patients with specific lymphoma subtypes, especially for patient populations that are at high risk. Therefore, exploration of the pharmacokinetic behaviour of rituximab in these patients is necessary to allow the optimal dosing.

Thus, a prospective pharmacokinetics study was conducted in patients with 6 uncommon lymphoma subtypes to more closely examine the concentration-outcome relationship and the related influencing factors to obtain evidence that can be used to individualize rituximab regimens.

## Materials and Methods

### Patients and Treatment Schedules

From 1 October 2016, to 28 February 2021, 51 patients were enrolled in a study conducted at Sun Yat-sen University Cancer Center, the largest cancer centre in South China. The inclusion criteria were age 18 years or older, previously untreated and histologically confirmed CD20^+^ Burkitt lymphoma, mixed B-cell lymphoma, high-grade B-cell lymphoma, MCL, MALT or unclassifiable B-cell lymphoma, and receiving first-line treatment with rituximab in combination with chemotherapy according to National Comprehensive Cancer Network (NCCN) guidelines. All patients received triweekly rituximab at the standard dose of 375 mg/m^2^ administered as an intravenous infusion. Clinical response was evaluated according to the Revised Response Criteria for Malignant Lymphoma (Cheson et al., 2007). The study was approved by the ethics committee of the Sun Yat-sen University Cancer Center, and all patients provided informed consent. This study was registered in the Chinese Clinical Trial Registry as ChiCTR1800017001 (http://www.chictr.org.cn/index.aspx).

### Blood Sampling

On the day of treatment with rituximab, samples were obtained immediately before and 0–3 h after each infusion. Plasma levels of rituximab and human antibodies against rituximab were determined through a solid phase enzyme-linked immunosorbent assay (ELISA), as previously described (Liu et al., 2020).

The blood samples used for CD19^+^ B-cell counts were collected before the first infusion treatment and analysed using a flow cytometry procedure as a surrogate marker for CD20^+^ B-cells, since their expression mirrors CD20 expression.

### Statistical Methods

All data were analysed using the SPSS Statistics 24.0 software package (SPSS Inc., Chicago, IL, United States). The nonparametric ANOVA Kruskal-Wallis test or Games-Howell test was used to compare concentrations among the patient groups. Comparisons between groups were analysed using T tests. Probability values of 0.05 or less were considered significant.

## Results

### Rituximab Concentrations in Different Lymphoma Subtypes

A total of 51 newly diagnosed patients were enrolled: 5 patients were diagnosed with MCL, 14 patients with non-gastric MALT, 6 patients with high-grade B-cell lymphomas, 4 patients with unclassifiable B-cell lymphoma (intermediate between DLBCL and classical Hodgkin’s), 11 patients with Burkitt lymphoma and 11 patients with mixed B-cell lymphoma (9 with DLBCL mixed with FL, and 2 with DLBCL mixed with MALT). The characteristics of the patients at baseline were shown in Table 1. All patients had normal liver and renal function.

**TABLE 1:** Summary of patients’ characteristics at baseline.

Subtype	MALT	MCL	Unclassifiable	Double-hit	NOS	Burkitt	Mixed B-cell
Number	14	5	4	3	3	11	11
Age (years)	59 (26–77)	61 (51–68)	30 (18–39)	42 (38–49)	45 (24–55)	36 (19–65)	63 (33–73)
Age (years) > 60	7 (50%)	4 (80%)	0	0	0	3 (27.27%)	6 (54.55%)
Sex, male	7 (50%)	4 (80%)	3 (75%)	2 (66.67%)	2 (66.67%)	9 (81.82%)	7 (63.63%)
BMI	21.64 (17.67–24.57)	21.30 (20.05–33.46)	22.58 (20.96–25.14)	20.55 (20.24–23.78)	21.94 (16.33–23.18)	20.31 (17.94–29.41)	22.86 (18.19–29.38)
BSA	1.54 (1.35–1.82)	1.67 (1.42–2.29)	1.70 (1.50–1.90)	1.63 (1.44–1.83)	1.60 (1.44–1.74)	1.65 (1.32–2.01)	1.73 (1.23–2.03)
LDH (U/L)	160.90 (135.10–304.60)	195.70 (154.20–353.60)	302.85 (168–347.20)	452.6 (379.90–666.50)	631.50 (198.40–900.40)	199.10 (92.60–1892.30)	173.30 (115.70–1860.50)
Hemoglobin (g/L)	128 (71–171)	124 (75–150)	139 (93–150)	127 (122–127)	113 (67–147)	127 (88–164)	2.11 (1.28–4.22)
β2-MG (mg/L)	1.85 (1.30–4.11)	2.43 (1.53–6.12)	2.23 (1.25–7.60)	1.39 (1.24–7.14)	2.43 (1.82–3.53)	1.48 (1.35–2.82)	126 (100–171)
ESR (mm/h)	19 (1–60)	22 (8–75)	7 (2–101)	10 (6–41)	70 (9–72)	21 (4–77)	17 (2–72)
Platelet (*10^9/L)	235 (154–543)	236 (191–270)	290 (203–400)	310 (133–468)	417 (206–418)	288 (185–367)	252 (151–475)
Stage IV	5 (35.71%)	3 (60%)	2 (50%)	3 (100%)	2 (66.67%)	2 (18.18%)	4 (36.36%)
CD19^+^	10.23 (1.22–27.70)	25.31 (6.23–67.48)	4.51 (2.10–13.10)	18.2 (16.70–18.50)	15.02 (6.64–19.86)	14.80 (4.60–20.70)	10.00 (5.00–33.30)
Bone marrow involvement	1 (7.14%)	3 (60%)	0	1 (33.33%)	0	1 (9.09%)	1 (9.09%)

All continuous values are reported as the median (minimum–maximum), categories are reported as numbers (percentages). MALT, marginal zone lymphoma; MCL, mantle cell lymphoma; Unclassifiable, unclassifiable B-cell lymphoma (intermediate between DLBCL and classical Hodgkin’s); “Double-hit”, High-grade B-cell lymphomas with translocations of MYC and BCL2 and/or BCL6; NOS, high-grade B-cell lymphomas appear blastoid or cases intermediate between DLBCL and Burkitt lymphoma, but which lack an MYC and BCL2 and/or BCL6 rearrangement. BMI, body mass index; BSA, body surface area; LDH, lactate dehydrogenase; β2-MG, β2-microglobulin; ESR, erythrocyte sedimentation rate; CD19^+^, the baseline proportion of CD19^+^ cells relative to the total number of lymphocytes in peripheral blood.

Rituximab concentrations were measured before and after each infusion in patients with different lymphoma subtypes. The peak concentrations of rituximab ranged from 155 to 346 μg/ml, and there was no significant difference in the peak concentrations among patients with different lymphoma subtypes or among patients with different treatment responses.

The rituximab trough concentrations in all patients in each cycle were shown in Table 2. Wide variations were observed in the first cycle trough concentration (C_1-trough_) among these patients; those concentrations ranged from 1.16 to 55.52 μg/ml (Table 2). Patients with “double-hit” lymphomas had the lowest rituximab C_1-trough_ (median, 4.36 μg/ml), followed by patients with MCL (10.28 μg/ml), patients with unclassifiable B-cell lymphoma (17.99 μg/ml) and patients with MALT (19.17 μg/ml). The median rituximab C_1-trough_ in the other groups was greater than 20 μg/ml, and patients with Burkitt lymphoma had the highest C_1-trough_ (26.32 μg/ml).

**TABLE 2:** Rituximab trough concentration (µg/ml) for patients with different lymphoma subtypes in each cycle.

Cycle	DLBCL/FL or MALT (mixed)	Burkitt	Unclassifiable	MALT	MCL	Double-hit	NOS
1	25.46 (20.27–36.34)	26.32 (17.65–48.49)	17.99 (17.44–21.34)	19.17 (2.96–55.52)	10.28 (1.16–41.20)	4.36 (3.12–4.54)	20.43 (12.95–30.49)
2	36.59 (24.36–57.62)	35.54 (19.12–57.75)	36.99 (28.47–40.96)	37.16 (3.12–58.88)	22.86 (5.44–45.46)	6.10 (6.09–7.58)	42.35 (38.56–47.78)
3	47.83 (28.06–66.06)	48.89 (36.87–70.12)	45.97 (35.22–52.14)	48.88 (29.17–60.21)	35.20 (22.28–48.87)	15.54 (11.78–25.58)	45.58 (40.33–56.84)
4	58.40 (36.74–76.43)	54.77 (35.50–78.89)	50.73 (50.68–65.60)	50.66 (35.35–61.37)	42.32 (28.82–52.33)	22.35 (11.68–28.56)	52.36 (50.22–55.02)
5	62.23 (40.60–86.15)	61.06 (41.09–80.12)	61.44 (41.58–70.26)	60.02 (39.16–78.88)	56.78 (46.44–60.44)	26.94 (24.48–30.05)	58.89 (49.78–60.66)

DLBCL, diffuse large B-cell lymphoma; FL, follicular lymphoma; Unclassifiable, unclassifiable B-cell lymphoma (intermediate between DLBCL and classical Hodgkin’s); MALT, marginal zone lymphoma; MCL, mantle cell lymphoma; “Double-hit”, High-grade B-cell lymphomas with translocations of MYC and BCL2 and/or BCL6; NOS, high-grade B-cell lymphomas appear blastoid or cases intermediate between DLBCL and Burkitt lymphoma, but which lack an MYC and BCL2 and/or BCL6 rearrangement. Data are median (range).

A steady increase in the trough concentration of rituximab was observed in subsequent cycles for all patients, and the concentrations eventually reached similar levels, except in the case of patients with “double-hit” lymphoma, those patients maintained very low rituximab concentrations throughout the observation period (Table 2).

For all patients, concentrations of human-antibodies against rituximab in any cycle remained below the quantification limit.

### Mucosa-Associated Lymphoma

**FIGURE 1:**
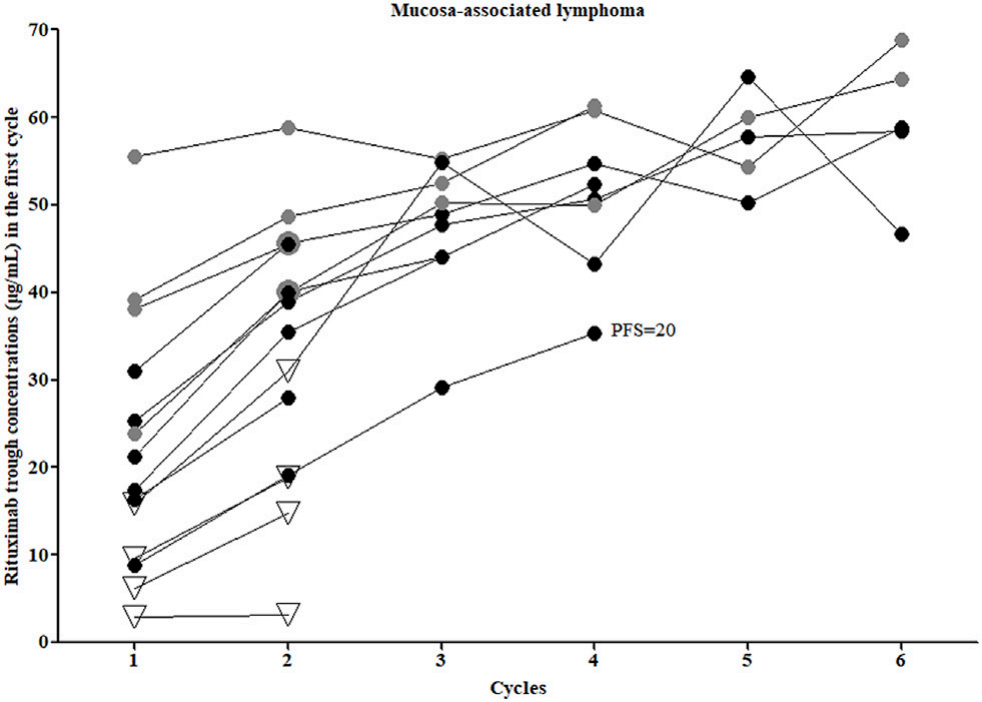
Rituximab concentrations and clinical outcomes of mucosa-associated lymphoma patients. Grey dots, patients who received rituximab combined with bendamustine; all other patients were treated with rituximab combined with CHOP. Hollow triangles, patients who did not achieve a complete response.

Fourteen patients with MALT (non-gastric) were treated with 2–6 infusions of chemoimmunotherapy, and then surgical excision, radiotherapy, or maintenance therapy were performed, depending on the situation. Triweekly infusions of standard-dose rituximab were administered, and 4 patients received bendamustine in combination (represented by the grey dots in Figure 1), the other 10 patients received CHOP (Figure 1; Table 3). All patients treated with RB achieved a complete response (CR) and experienced no relapse as of the last follow-up. Of the 10 patients who received R-CHOP, 6 patients achieved a CR, one of these patients, who had a C_1-trough_ of 8.89 μg/ml subsequently relapsed involving the orbital adnexa within 2 years (PFS = 20 months). Four patients achieved partial remission (PR) or stable disease (SD) (represented by the hollow triangles in Figure 1), but in these patients no disease progression was observed after adjusting for treatment regimens. The C_1-trough_ was significantly higher in patients treated with RB (median, 38.59 μg/ml, range, 20.93–55.52 μg/ml) than in patients who received R-CHOP (median, 16.10 μg/ml, range, 2.96–25.33 μg/ml, *p* = 0.0029), despite the fact that the former group included a higher percentage of patients with stage IV disease (75 vs 20%). The C_1-trough_ in patients with CR (median, 23.27 μg/ml, range, 8.89–55.52 μg/ml) was superior to that in patients who did not achieve a CR (median, 7.92 μg/ml, range, 2.96–15.83 μg/ml, *p* = 0.0237).

**TABLE 3:** Rituximab concentrations, baseline characteristics and clinical outcomes of marginal zone lymphoma patients, mantle cell lymphoma patients, unclassifiable B-cell lymphoma (intermediate between DLBCL and classical Hodgkin’s) patients, high-grade B-cell lymphoma patients, Burkitt lymphoma patients and mixed B-cell lymphoma patients.

Marginal zone lymphoma (MALT)																																					
First-line regimen		Number of patients							Stage IV					CD19^+^							C_1-trough_(μg/mL)							CR	PR			SD		ORR			Recurrence
RB		4							75%					8.87 (1.22–10.05)							38.59 (20.93–55.52)							100%	0			0		100%			No
R-CHOP		10							20%					38.59 (1.70–27.70)							16.10 (2.96–25.33)							60%	30%			10%		90%			1 patient
Mantle cell lymphoma (MCL)																																					
Patient	First-line regimen					Age		Stage			CD19^+^				Ki-67				C_1-trough_ (μg/ml)					Response			Recurrence			PFS (months)						OS (months)	
1	R-CHOP/R-DHAP					68		1			8.40				90%				22.16					CR			No			40						40	
2						61		2			6.23				15%				41.20					CR			No			26						26	
3						61		4			25.31				20%				10.28					PR			No			27						27	
4						51		4			47.12				20%				3.44					SD			No			26						26	
5						61		4			67.48				60%				1.16					PD						3						14	
Unclassifiable B-cell lymphoma																																					
Patient	First-line regimen					Age		Stage				CD19^+^				C_1-trough_ (μg/ml)							Response			Recurrence			PFS (months)						OS (months)		
1	R-CHOP					34		4				13.1				21.34							CR			No			33						33		
2						39		4				5.81				18.50							PD						3						29		
3						17		3				2.1				17.47							CR			No			43						43		
4						25		2				3.2				17.44							CR			No			31						31		
High-grade B-cell lymphomas																																					
Patient	Classification			First-line regimen										Age		Stage			LDH (U/L)			CD19^+^			C_1-trough_ (μg/ml)		Response		Recurrence				PFS (months)				OS (months)
1	NOS			R-CODOX-M										24		1			198.4			19.86			30.49		CR		No				31				31
2	NOS			R-CODOX-M										55		4			631.5			6.64			20.43		CR		No				17				17
3	NOS			R-CODOX-M										45		4			900.4			15.02			12.95		CR		No				21				21
4	Double-hit			R-CODOX-M/R-IVAC										49		4			452.6			16.7			4.36		PD						1				6
5	Double-hit			R-CODOX-M/R-IVAC										38		4			379.9			18.2			4.54		PD						3				15
6	Double-hit			R-CODOX-M/R-IVAC										42		4			666.5			18.5			3.12		CR		Yes				6				7
Burkitt lymphomas																																					
Patient	First-line regimen						Age			Stage			LDH (U/L)					CD19^+^				C_1-trough_ (μg/ml)					Response			PFS (months)						OS (months)	
1	R-CODOX-M/R-IVAC						24			1			227.7					10.80				26.32					CR			30						30	
2	R-CODOX-M						61			1			163.5					9.3				28.81					CR			37						37	
3	R-CODOX-M						32			1			92.6					20.7				25.45					CR			34						34	
4	R-CDOP-HD MTX						65			1			96.7					12.8				37.72					CR			43						43	
5	R-CODOX-M/R-IVAC						36			2			190.5					17.81				23.77					CR			42						42	
6	R-CODOX-M						19			2			128.2					14.8				19.69					CR			49						49	
7	R-CDOP-HD MTX						45			3			251.2					4.6				32.87					CR			42						42	
8	R-CDOP-HD MTX						29			3			199.1					18.4				17.65					CR			42						42	
9	R-CDOP-HD MTX						60			3			440.2					16.4				19.91					PD			1.4						12	
10	R-CODOX-M/R-IVAC						15			4			1892.3					20.5				36.67					CR			52						52	
11	R-CODOX-M/R-IVAC						47			4			766.5					11.41				48.49					CR			18						18	
Mixed B-cell lymphoma																																					
Outcome			N		First-line regimen							Age					Stage Ⅳ			CD19^+^						C_1-trough_ (μg/ml)					PFS (months)					OS (months)	
CR and no recurrence			9		R-CHOP							49 (33–70)					11.11%			10.30 (5.00–33.30)						28.10 (20.27–36.34)					37 (28–40)					37 (28–40)	
Recurrence after a CR			1		R-CHOP							73					Yes			8.5						21.78					36					41	
Continuous progression			1		R-CHOP							66					Yes			7.6						22.41					3					9	

N, Number of patients; CD19^+^, the baseline proportion of CD19^+^ cells relative to the total number of lymphocytes in peripheral blood; C_1-trough_, rituximab trough concentrations in the first cycle; CR, complete response; PR, partial response; SD, stable disease; PD, progressive disease; ORR, overall response rate; PFS, progression-free survival; OS, overall survival; LDH, lactate dehydrogenase; NOS, high-grade B-cell lymphomas that appeared blastoid or cases intermediate between DLBCL and Burkitt lymphoma that lacked MYC and BCL2 and/or BCL6 rearrangements; Double-hit, high-grade B-cell lymphomas with translocations of MYC and BCL2 and/or BCL6; Data are median (range).

### Mantle Cell Lymphoma

Five patients with MCL received triweekly R-CHOP/R-DHAP as a first-line treatment regimen, and a positive correlation between the rituximab C_1-trough_ and outcomes was observed (Table 3). Two patients who achieved a CR had high C_1-trough_ (41.20 and 22.16 μg/ml). The remaining three patients achieved PR, SD and had progressive disease, respectively, with a decreasing C_1-trough_ of 10.28, 3.44, and 1.16 μg/ml. The patient with an extremely low C_1-trough_ of 1.16 μg/ml continued to progress after subsequent salvage treatment and died 14 months later. In the other two patients, the disease was controlled after the addition of BTK inhibitors to the treatment regimen.

### Unclassifiable B-Cell Lymphoma

Four patients with unclassifiable B-cell lymphoma received R-CHOP as initial therapy, and the C_1-trough_ of rituximab of these patients were similar, ranging from 17.44 to 21.34 μg/ml (Table 3). One patient failed first-line treatment with the rituximab C_1-trough_ of 18.50 μg/ml, but CR was achieved following the addition of brentuximab, and similar to the other patients, this patient has not relapsed so far.

### High-Grade B-Cell Lymphomas

Six patients with high-grade B-cell lymphomas were included in this study (Table 3). Three patients with “double-hit” lymphomas had a significantly lower rituximab C_1-trough_ than the three not otherwise specified (NOS) patients (*p* = 0.0276, Table 3). All “double-hit” patients progressed rapidly and had a very short OS, even one patient achieved Deauville 3 briefly during treatment. The three NOS patients all achieved a CR and had no relapse as of the last follow-up.

### Burkitt Lymphoma

The rituximab C_1-trough_ for patients with Burkitt lymphoma ranged from 17.65 to 48.49 μg/ml (Table 3). Of the 11 patients, four patients had lactate dehydrogenase (LDH) levels higher than 250 U/L before treatment. However, among these patients, only one failed first-line treatment, and then the disease rapidly became uncontrolled (OS was 12 months) with a C_1-trough_ of 19.91 μg/ml. The rituximab C_1-trough_ values of the other 3 patients were 32.87, 36.67, and 48.49 μg/ml, respectively, and all of them achieved a CR with first-line treatment and have not relapsed so far.

### Mixed B-Cell Lymphoma

The rituximab C_1-trough_ for 9 patients diagnosed with DLBCL mixed with FL ranged from 20.27 to 34.47 μg/ml, and two patients diagnosed with DLBCL mixed with MALT had a rituximab C_1-trough_ of 20.38–36.34 μg/ml, respectively (Table 3). The median C_1-trough_ for these patients was 25.46 μg/ml. One patient (DLBCL mixed with FL) progressed rapidly with a C_1-trough_ of 22.41 μg/ml, and OS was short as 9 months. Another patient (DLBCL mixed with FL) recurred 36 months after the first-line treatment with a C_1-trough_ of 21.78 μg/ml.

### Concentration-Outcome Relationship and Influencing Factors

**FIGURE 2:**
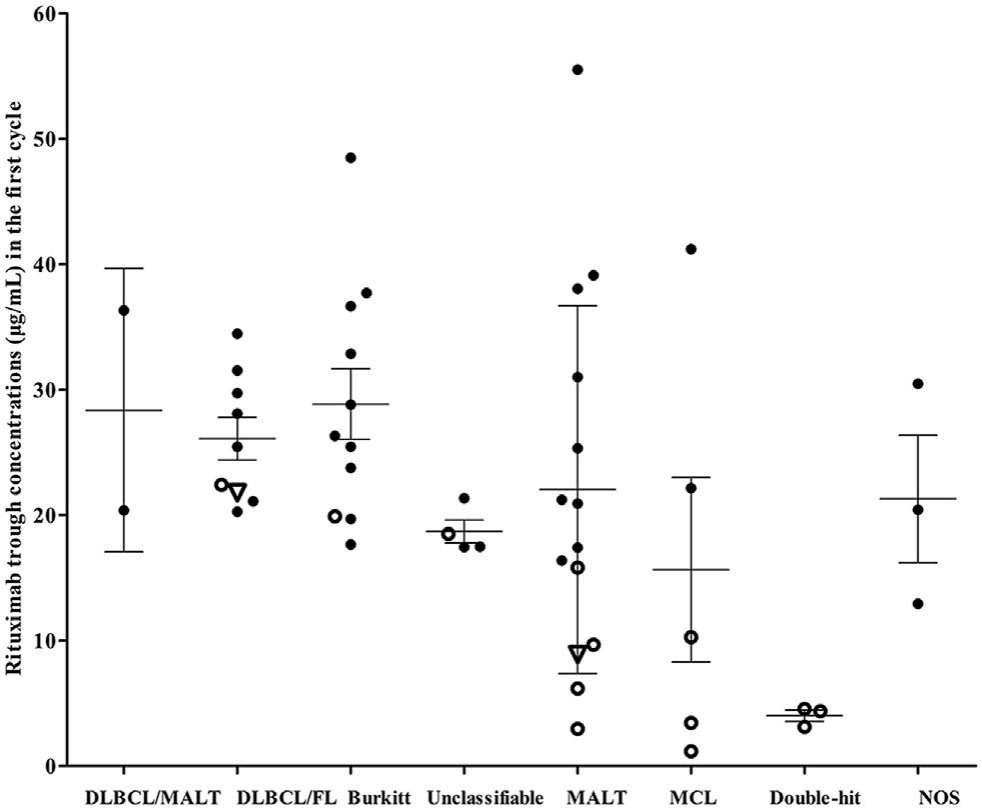
Rituximab concentrations and clinical outcomes of patients with different lymphoma subtypes. Hollow dots, patients who failed first-line treatment; hollow triangles, patients who relapsed after achieving a complete response. DLBCL, diffuse large B-cell lymphoma; FL, follicular lymphoma; Unclassifiable, unclassifiable B-cell lymphoma (intermediate between DLBCL and classical Hodgkin’s); MALT, marginal zone lymphoma; MCL, mantle cell lymphoma; “Double-hit”, High-grade B-cell lymphomas with translocations of MYC and BCL2 and/or BCL6; NOS, high-grade B-cell lymphomas appear blastoid or cases intermediate between DLBCL and Burkitt lymphoma, but which lack an MYC and BCL2 and/or BCL6 rearrangement.

Among the patients who were treated, patients who achieving a CR with first-line treatment had significantly higher mean C_1-trough_ than patients who did not achieve a CR (38.41 ± 14.13 μg/ml vs 15.49 ± 8.80 μg/ml, *p* = 0.0029, Figure 2). Two patients relapsed after achieving a CR with first-line treatment with a rituximab C_1-trough_ of 8.89 μg/ml (MALT) and 21.78 μg/ml (mixed B-cell lymphoma), respectively. One patient with “double-hit” lymphoma and a rituximab C_1-trough_ value of 3.12 μg/ml achieved Deauville 3 briefly, but progressed rapidly thereafter and had a very short OS. Five patients continued to progress from the start of treatment, and all of these patients had short OS, their rituximab C_1-trough_ values were 4.36 μg/ml (“double-hit” lymphoma), 4.54 μg/ml (“double-hit” lymphoma), 1.16 μg/ml (MCL), 19.91 μg/ml (Burkitt lymphoma) and 22.41 μg/ml (mixed B-cell lymphoma). Obviously, extremely low rituximab C_1-trough_ or lower value in its own subgroup was associated with adverse consequences.

The low rituximab concentration could be partially explained by the presence of a high tumour burden (advanced stage). To gain more insight into the factors that may influence the rituximab C_1-trough_, sex, age, body surface area (BSA), body mass index (BMI), initial bone marrow infiltration, bone metastases, bulky masses (>7 cm), basal circulating CD19^+^ B-lymphocyte counts, Ki-67, LDH, beta 2-microglobulin (β2-MG), and erythrocyte sedimentation rate (ESR) were analysed by linear regression. The circulating CD19^+^ B lymphocyte count was significantly negatively associated with the rituximab C_1-trough_ (C_1-trough_ = 28.78–0.44*CD19^+^), and the adjusted R square was 16.15%.

The median basal circulating CD19^+^ B lymphocyte counts were 25.31 (range, 6.23–67.48), 18.2 (range, 16.70–18.50), 15.02 (range, 6.64–19.86), 14.80 (range, 4.60–20.70),10.23 (range, 1.22–27.70), 10.00 (range, 5.00–33.30) and 4.51 (range, 2.10–13.10) for MCL, “double-hit” lymphoma, NOS high-grade B-cell lymphoma, Burkitt lymphoma, MALT, mixed B-cell lymphoma and unclassifiable B-cell lymphoma, respectively.

### Indolent Lymphomas Versus Aggressive Lymphomas

The studied groups of lymphomas were further separated into a “clinically indolent lymphomas” group (MALT and MCL classical type with ki-67 ≤ 30%) and a “clinically aggressive lymphomas” group (high-grade B-cell lymphomas, Burkitt lymphoma, unclassifiable B-cell lymphoma, mixed B-cell lymphoma and mantle cell lymphoma with ki-67 > 30%). The rituximab trough concentrations in these two groups in each cycle were shown in Table 4, the concentrations were similar in the two groups.

**TABLE 4:** Rituximab trough concentration (µg/ml) for patients with indolent lymphomas and aggressive lymphomas in each cycle.

Cycle	Indolent lymphomas	Aggressive lymphomas
1	17.41 (2.96–55.52)	21.97 (1.16–48.49)
2	35.45 (3.12–58.88)	35.35 (5.44–57.75)
3	48.32 (29.17–55.22)	45.50 (11.78–70.12)
4	49.42 (35.35–61.37)	50.45 (11.68–78.89)
5	58.87 (39.16–78.88)	56.72 (24.48–86.15)

Data are presented as the median (range). Indolent lymphomas included marginal zone lymphoma and mantle cell lymphoma classical type with ki-67 ≤ 30%. Aggressive lymphomas included high-grade B-cell lymphomas, Burkitt lymphoma, unclassifiable B-cell lymphoma (intermediate between DLBCL and classical Hodgkin’s), mixed B-cell lymphoma and mantle cell lymphoma with ki-67 > 30%.

**FIGURE 3:**
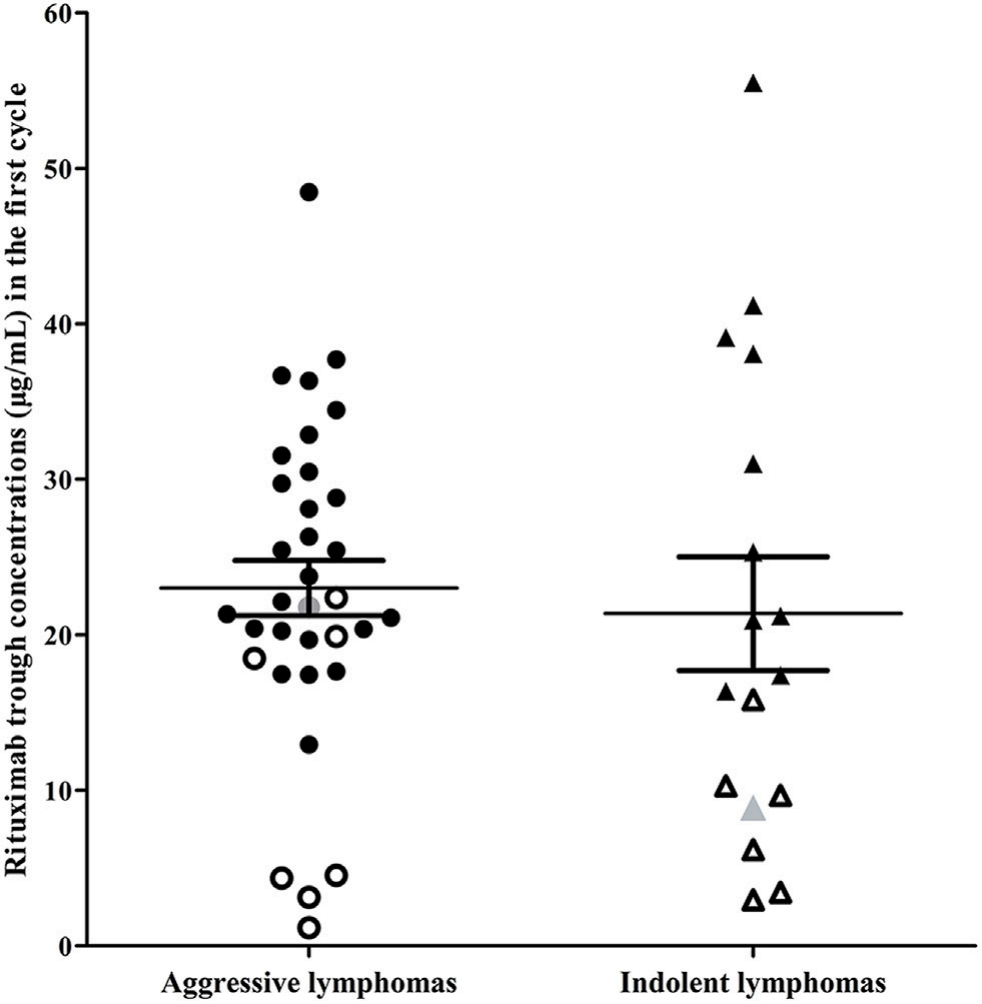
Rituximab concentrations and clinical outcomes of patients in the indolent lymphoma group and the aggressive lymphoma group. Indolent lymphomas (triangle) included marginal zone lymphoma and mantle cell lymphoma classical type with ki-67 ≤ 30%. Aggressive lymphomas (dot) included high-grade B-cell lymphomas, Burkitt lymphoma, unclassifiable B-cell lymphoma (intermediate between DLBCL and classical Hodgkin’s), mixed B-cell lymphoma and mantle cell lymphoma with ki-67 > 30%. Hollow dots or triangles, patients who failed first-line treatment; gray dot or triangle, patients who relapsed after achieving a complete response.

In the indolent lymphomas group, the C_1-trough_ in patients with a CR (median, 25.33 μg/ml, range, 8.89–55.52 μg/ml) was greater than that in patients who did not achieve a CR (median, 7.93 μg/ml, range, 2.96–15.83 μg/ml, *p* = 0.0001). A similar phenomenon was observed in the aggressive lymphomas group (CR, median, 25.45 μg/ml, range, 12.95–48.49 μg/ml versus non-CR, median, 4.54 μg/ml, range, 1.16–22.41 μg/ml, *p* < 0.0001). The rituximab concentrations and clinical outcomes of patients in the indolent and aggressive lymphomas groups were shown in Figure 3.

## Discussion

This study reported the pharmacokinetic properties of rituximab in patients with 6 different subtypes of lymphoma, all of which represent small groups of tumours, and to our knowledge, the rituximab pharmacokinetics in some subtypes have never been reported. Considerable variability in the initial rituximab trough concentration was observed in patients with different lymphoma subtypes, and a low C_1-trough_ was associated with adverse consequences, however, some high-risk factors appear to be balanced by the presence of a high C_1-trough_. Therefore, the dose of rituximab used to treat patients with different lymphoma subtypes should be tailored to the disease.

Based on our results, better clinical outcomes could be predicted in the presence of a higher rituximab C_1-trough_, and no adverse consequence happened for patients with a C_1-trough_ higher than 23 μg/ml (Figure 2). However, patients who failed the first-line treatment (15.49 ± 8.80 μg/ml) and relapsed after achieving a CR with first-line treatment (median, 8.89 μg/ml) and continued to progress and died (median, 4.54 μg/ml) all had a significantly low C_1-trough_. Moreover, the “double-hit” lymphoma and MCL are both characterized by unsatisfactory therapeutic effect, and patients with those two diseases all had lower C_1-trough_ (4.01 ± 0.77 μg/ml and 15.65 ± 16.45 μg/ml) was first observed in this study, suggesting that the inadequate dose of rituximab is probably an important cause of poor treatment outcomes.

In this study, no human-antibodies against rituximab were detected in patients with the studied lymphoma subtypes, and the low incidence of the development of antibodies against rituximab in lymphoma has also been reported in other studies (Berinstein et al., 1998).

The translocations of MYC and BCL2 and/or BCL6 in patients with high-grade B-cell lymphomas remained a significant independent predictor of a poorer PFS and OS (Oki et al., 2014; Petrich et al., 2014), but the standard of care for these patients has not been established. The limited available clinical trials (Petrich et al., 2014; Sun et al., 2015) suggested that treatment with intensive regimens such as DA-EPOCH-R, R-HyperCVAD or R-CODOX-M/IVAC resulted in superior CR and PFS compared to R-CHOP; however, OS was not significantly different among these approaches, and intensive regimens inevitably aggravated the adverse reactions. Novel strategies are urgently needed to improve outcomes in this high-risk patient population. In this study, extremely low rituximab C_1-trough_ were observed in three patients with “double-hit” lymphoma (3.12, 4.36, and 4.53 μg/ml). Since Strussmann et al. (2017) and Murawski et al. (2014) both reported that DLBCL patients with a poor prognosis receiving initial dense-dose rituximab had a more promising response than those receiving the standard regimen, increasing the first dose of rituximab is likely to represent a new therapeutic approach that may greatly benefit high-grade B-cell lymphoma patients with specific translocations.

MCL comprises approximately 3% of all newly diagnosed cases of non-Hodgkin’s lymphomas (NHLs) (Teras et al., 2016). It has been suggested that the Ki-67 proliferation index remains an important prognostic marker independent of clinical prognostic factors, and a low Ki-67 (<30%) was associated with a more favourable prognosis (Determann et al., 2008; Schaffel et al., 2010). Of the 5 MCL patients in this study, 3 patients had Ki-67 below 30% with rituximab C_1-trough_ of these patients were 41.20, 10.28, and 3.44 μg/ml. Only the patient with a C_1-trough_ of 41.20 μg/ml achieved a CR and did not experience relapse. The other two patients both progressed on first-line therapy, and the disease remained uncontrolled until BTK inhibitors were included in the treatment. Of two patients with high Ki-67 levels of 90 and 60%, the patient with a higher C_1-trough_ (22.16 μg/ml, Ki-67, 90%) achieved a CR after 2 courses of treatment and had not relapsed as of the last follow-up (40 months); however, the other patient with a lower C_1-trough_ (1.16 μg/ml, Ki-67, 60%) progressed after 4 courses of treatment, and the OS was 14 months. Despite the small number of cases in this study, the results suggest that the monitoring of rituximab C_1-trough_ could potentially aid in the individualized management of MCL patients.

Marginal zone lymphoma is an indolent lymphoma that comprises 5–10% of all NHLs (Teras et al., 2016). The NCCN B-Cell Lymphomas Panel recommended BR and R-CHOP as the preferred regimens for first-line therapy. In this study, for the five patients with the lowest concentration (all below 20 μg/ml), four patients failed to achieve a CR after first-line therapy, and the remain one relapsed within 2 despite achieving a CR. Moreover, patients with RB had significantly higher C_1-trough_ than patients with R-CHOP, although the RB group included a higher proportion of patients with stage IV disease. These findings are consistent with the results of some clinical trials that reported that BR was superior to R-CHOP in terms of overall response rates (ORRs), CR or PFS (Rummel et al., 2013; Flinn et al., 2014). Therefore, an increase in the initial dose of rituximab or in combination with bendamustine was recommended for patients with high-risk factors.

Of the 11 patients with mixed B-cell lymphoma, three patients had stage IV disease, and two of them had progression or relapse, those two patients had lower rituximab C_1-trough_ of 21.78–22.41 μg/ml. Another stage IV patient had a PFS 39 of months, no relapse, and with a C_1-trough_ of 31.53 μg/ml. Higher initial concentrations of rituximab appeared to be a protective factor for these patients.

Burkitt lymphoma is a rare and highly aggressive B-cell lymphoma, but is curable in a significant subset of patients when treated with dose-intensive, multiagent chemotherapy regimens (Wasterlid et al., 2013; Oosten et al., 2018). Burkitt lymphoma patient had the highest rituximab C_1-trough_ (median, 26.32 μg/ml) in this study. In these highly aggressive lymphomas, as in DLBCLs, the serum LDH level has prognostic significance (Perry et al., 2013). Four patients in this study had significantly elevated LDH (>250 U/L) and advanced-stage, only one patient failed to achieve a CR and had a poor survival outcome with a lower rituximab C_1-trough_ of 19.91 μg/ml, the other three patients all had a rituximab C_1-trough_ higher than 30 μg/ml and achieved durable remission. It appeared that a higher rituximab C_1-trough_ balanced out some risk factors and thereby improve clinical responses.

The pharmacokinetics of rituximab in patients with uncommon subtypes of lymphoma, especially MCL, have been reported by several groups. Mangel et al. (2003) conducted a pharmacokinetic study of 26 lymphoma patients (20 FL, 6 MCL) in an autologous stem cell transplant setting and found that there was a trend towards lower rituximab levels in patients with MCL compared with patients with FL at almost all time points. Tran et al. (2010) reported rituximab concentrations in 8 patients (2 DLBCL, 4 FL, 1 chronic lymphoid leukaemia and 1 MCL), the patient with MCL received a 4-weekly schedule of R-PECC, and had the lowest serum levels of rituximab, even 83 days after the last rituximab infusion, a much lower rituximab serum level of 20.2 μg/ml was measured. Inter-individual variations in plasma concentrations of rituximab in B cell non-Hodgkin’s lymphoma patients were also found in Yonezawa’s study (Yonezawa et al., 2019), that study included 20 patients (9 FL, 7 DLBCL, 2 FL/DLBCL, 1 MCL and 1 intravascular large B cell lymphoma), the MCL patient showed rapid rituximab clearance, approximately 4-fold higher than the clearance exhibited by the other patients.

A similar phenomenon was observed in the current study in that the MCL patient group had much lower rituximab concentrations (median, 10.28 μg/ml) than the patients in the other lymphoma subtype groups, and this level was also lower than that of FL patients (median, 18.49 μg/ml) in our previous study (Liu et al., 2020), who were subjected to the same dosing interval. Mangel et al. (2003) postulated that perhaps more active non-specific clearance accounts for the lower levels of rituximab observed in patients with MCL. Another group, patients with “double-hit” lymphoma, had the lowest rituximab concentration (median, 4.36 μg/ml) observed in this study, and the reason for this was also unclear. Taken together, the results of these studies suggested that patients with different lymphoma subtypes may actually require different rituximab doses or different dosing schedules to achieve optimal results.

In Mangel’s study (Mangel et al., 2003), no correlation was observed between serum rituximab levels and clinical benefit in 6 MCL patients in an autologous stem cell transplant setting. However, in the induction treatment setting, a statistically significant correlation was found between the serum rituximab concentration and clinical response in a number of studies for patients with DLBCL (Tout et al., 2017; Liu et al., 2021) and FL (Liu et al., 2020), which were consistent with the results in this study. The different treatment setting could be the main reason for this contradicts.

Almost all clinical studies in which rituximab serum levels have been evaluated have demonstrated that the observed large interindividual variability in rituximab serum levels could be related to the “target burden” (Cartron et al., 2007; Golay et al., 2013; Wang et al., 2020). It is not surprising that the baseline CD19^+^ lymphocyte count was found to be inversely correlated with the rituximab level, a higher concentration of rituximab would be needed to neutralize the high amount of circulating B cells in the peripheral blood. The observation indicated that differences in the levels of target expression, tumor physiology, and disease characteristics may all influence rituximab exposure and dosage selection.

A prospective study was conducted in patients with 6 uncommon subtypes of lymphoma to explore the concentration-outcome relationship of rituximab. Low rituximab C_1-trough_ was associated with adverse consequences, however, some high-risk factors appear to be balanced by the presence of high C_1-trough_. Extremely low C_1-trough_ in patients with “double-hit” lymphoma and MCL was observed in this study, suggesting the traditional doses of rituximab are inadequate for the treatment of these two diseases. Increasing the initial rituximab dose according to the specific disease, the presence of high-risk factors and even the baseline CD19^+^ lymphocyte count will be new methods to optimize therapeutic regimens for different lymphoma subtypes.

## Data Availability

The original contributions presented in the study are included in the article/Supplementary Material, further inquiries can be directed to the corresponding authors.
